# Morphological and genetic characterization of the broad mite *Polyphagotarsonemus latus* Banks (Acari: Tarsonemidae) from two Mexican populations

**DOI:** 10.1371/journal.pone.0266335

**Published:** 2022-04-21

**Authors:** Vivian Ovando-Garay, Rebeca González-Gómez, Eugenia Zarza, Alfredo Castillo-Vera, Martha Elena de Coss-Flores

**Affiliations:** 1 Departamento de Agricultura, Sociedad y Ambiente, Ecología de Artrópodos y Manejo de Plagas, El Colegio de la Frontera Sur, Tapachula, Chiapas, México; 2 Investigadoras e Investigadores por México, Consejo Nacional de Ciencia y Tecnología, Ciudad de México, México; 3 Departamento de Ciencias de la Sustentabilidad. Biotecnología Ambiental. El Colegio de la Frontera Sur, Tapachula, Chiapas, México; 4 MEKOSS, Empresa Innovadora del Sector Salud y Agropecuaria, Tapachula, Chiapas, México; Nanjing Agricultural University, CHINA

## Abstract

*Polyphagotarsonemus latus* Banks is considered a polyphagous pest of diverse agricultural and ornamental crops of global economic significance. Its distribution, host range, variety of symptoms, morphological differences, chaetotaxy and several ontogeny reports have advanced the idea of *P*. *latus* as a species complex. Correct pest identification leads to suitable control treatment. Therefore, the objective of this study was the identification of mites collected in two different geographic regions in Mexico (Chiapas and Guanajuato) that had been tentatively designated as *Polyphagotarsonemus* sp. Biometric differences on the morphology of adults as well the genetic variability were determined by taxonomical and molecular (mitochondrial COI gene) characterization techniques. The identity of the mites from both populations was confirmed as *P*. *latus* based on taxonomic characters. Biometric parameter variations were found between both populations (70.58% and 53.84% for females and males, respectively). The average sequenced fragment size was 447 bp (both populations). A homology search against six *P*. *latus* sequences available in the GenBank database revealed that sequence KM580507.1 (from India) shows 83.0–86.41% and 99.26–99.52% similarity with the sequences from Guanajuato and Chiapas, respectively. Molecular data indicated a significant divergence between the populations. The genetic distance demonstrates the population from Chiapas has a higher genetic correspondence (0.010) to the sequence from India (KM580507.1) whereas the population from Guanajuato is more distant (0.191). The genetic distance between the populations of this study and other GenBank sequences is even larger. We consider our results strengthen the hypothesis of *P*. *latus* consisting of a species-complex. However, it is essential to extend the study to other regions including its country of origin (Sri Lanka), and to include ultrastructural features.

## Introduction

The phytophagous broad mite *Polyphagotarsonemus latus* Banks (Acari: Tarsonemidae) is a cosmopolitan pest [1]. It is prevalent in tropical and subtropical regions but has been recorded in temperate areas as well. The broad mite has been reported in Australia, Asia, Africa, Europe, North and South America, and the Pacific Islands [1]. This phytophagous mite has a wide range of hosts belonging to more than 60 botanical families [1]. It is considered a significant polyphagous pest of global agroeconomic importance which especially affects citrus production—sweet orange (*Citrus x sinensis* [L.] Osbeck), bitter orange (*Citrus x aurantium* L.), Cleopatra mandarin (*Citrus reshni* Hort. Ex Tanaka) [2,3] but also various species of chilli (*Capsicum annuum* L., *Capsicum frutescens* L.), among several crops [4–7].

*P*. *latus* affects plant tissues that are in active growth (leaves, flowers, shoots, and others) and deposits its eggs on the lower surface of leaves. Its life cycle is very short (four to five days) and largely depends on relative humidity and temperature [8–10]. Furthermore, broad mites often go undetected at the outset of an outbreak due to their small size (0.1 to 0.2 mm long). Their presence is recognized only at the moment that infested plants already begin to exhibit signs of damage [2,4,11,12].

Injury induced by *P*. *latus* can manifest a wide variety of symptoms, which some authors attribute to the toxicity of the mite’s saliva and host plant reaction mechanisms. Symptoms include leaf bronzing and misshapen fruits, distorted flowers and producing multiple buds [13], and the curling of leaf margins. Injury, however, may be mistaken for virus or herbicide damage [1], nutritional deficiency or physiological disorders [14]. However, this symptom variability may be caused by a species-complex [15] or feeding ecotypes [14]. Also, descriptions of external morphology, chaetotaxy and ontogeny of *P*. *latus* from Mexico, Germany, Cuba and Costa Rica, have demonstrated considerable biometric variability in this species [8,16–18].

In general, phytophagous mites constitute one of the principal threats to agriculture and their treatment with acaricides entails a serious risk to ecosystems worldwide. Taxonomy is essential for the nomenclature, identification and classification of organisms, and therefore represents a basic component of pest control programs. Therefore, the success or failure of control measures will depend in the first place on correct pest species identification [19].

Today, integrative mite identification strategies are adopted, including molecular markers combined with scanning electron microscope (SEM) for morphological analyses. In the current investigation, mites collected from two regions in Mexico (Chiapas and Guanajuato) were tentatively designated as *Polyphagotarsonemus* sp. Their identity was confirmed as *Polyphagotarsonemus latus* by taxonomical and molecular characterization. This was accomplished by a biometric analysis of the morphology of adults, and by a phylogenetic analysis of the mitochondrial COI gene (cytochrome c oxidase subunit I).

## Materials and methods

### Biological material

Mite specimens were collected from two populations, one from Southern and one from Central Mexico. In Southern Mexico, mites were collected from *Tecoma stans* [20] Juss. ex Kunth (Bignonacea) trees in Tapachula, Chiapas (177 m.a.s.l., 14°54’00"N, 92°16’00"W). This evergreen tropical tree is native to Mexico, the Caribbean Islands, and the Southern United States [21–23] and is commonly known as trumpet flower in English, *tronadora* in Spanish, and *tecomaxochitl* in Nahuatl. In Central Mexico, mites were collected from a commercial greenhouse (Agrícola Sabora) bell pepper (*Capsicum annuum* L., Solanaceae) plantation in Pénjamo, Guanajuato (1770 m.a.s.l., 20°25’52"N, 101°43’20"W). Specimens from each population were collected in July 2020 and transferred into Eppendorf tubes containing 100% alcohol, transported to the laboratory (*Salud Forestal de El Colegio de la Frontera Sur*, Tapachula, Chiapas) and stored at 4°C for analysis.

### Morphological and biometric study

The mite species was identified based on morphological characteristics under a dissecting microscope (Carl Zeiss Stemi 2000C at 40x). The mites were placed in a staining plate (cavities: 16 mm diameter, 2.3 mm depth) and covered with 1% lactic acid (clearing medium) for one week. Next, they were mounted on glass slides using Hoyer’s medium: a mite was placed into a droplet of Hoyer’s medium on the centre of a microscopic slide and covered with a coverslip (1 x 1 cm). The slides were then placed in a drying oven at 45°C for 7 days. After the drying process, the coverslip was sealed with insulating paint. Each slide was labelled with origin (sampling site), host, collecting date and collector [24]. The slide-mounted mites were identified under a phase-contrast microscope (Carl Zeiss Axio Lab.A1 at 100x) according to the morphological criteria established by Lindquist (1986) [15] and Walter et al. [25]. A total of 88 adult specimens were examined—47 (25 females and 22 males) from Southern Mexico (Chiapas) and 41 (23 females and 18 males) from Central Mexico (Guanajuato).

**Fig 1:**
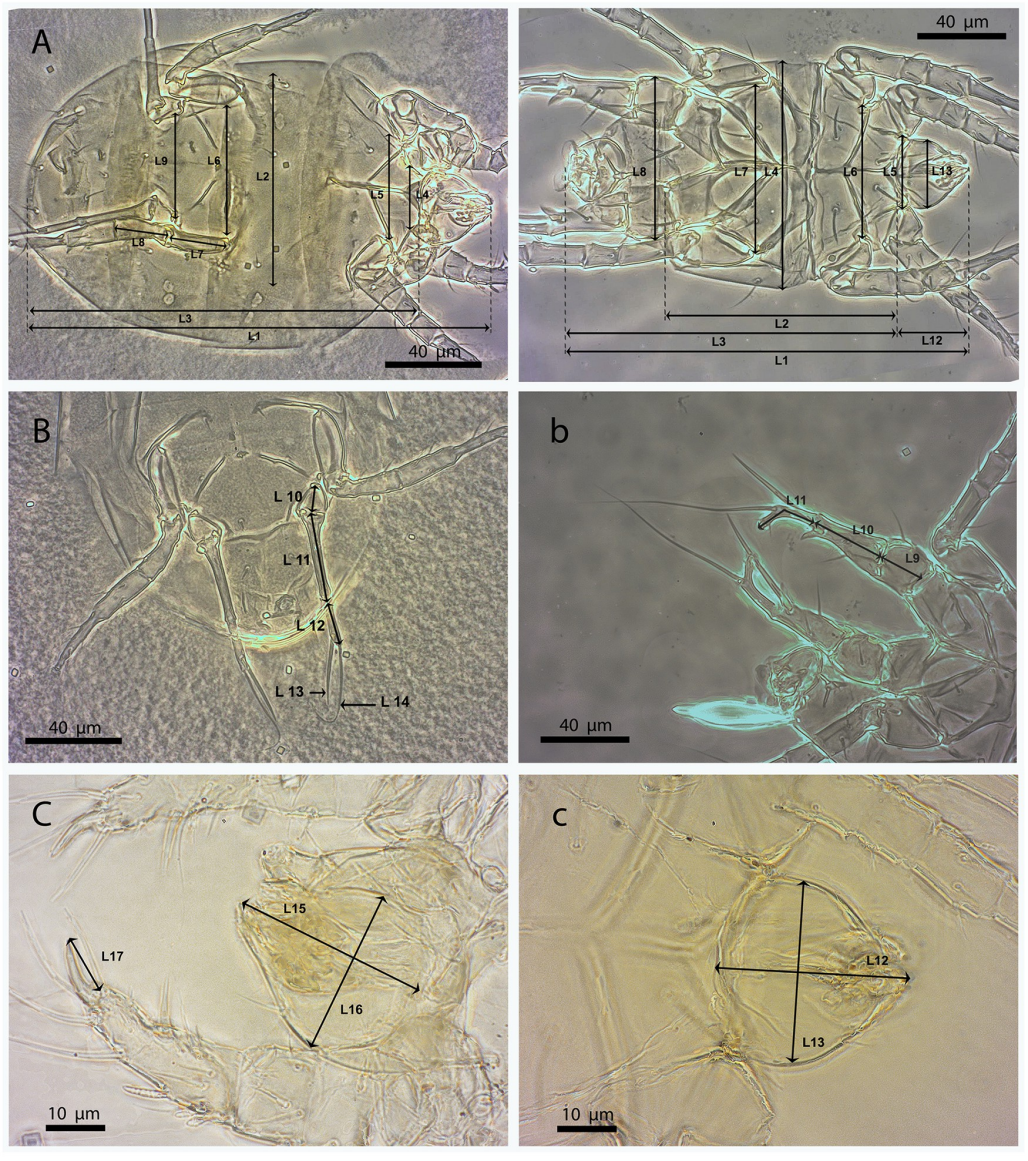
Biometric parameters evaluated in *Polyphagotarsonemus latus* (female and male). Females (A, B and C) and males (a, b and c). The key to the abbreviations can be found in Materials and methods (Morphological and biometric study).

A biometric analysis was performed following the criteria outlined by De Coss [16] for adult *P*. *latus* mites of the Soconusco region, Chiapas. For females, 17 parameters were examined: total length (L1), idiosome width (L2), idiosome length (L3), distance between trochanters I (L4), distance between trochanters II (L5), distance between trochanters III (L6), length of trochanter III (L7), length of femorogenu III (L8), distance between trochanters IV (L9), length of trochanter IV (L10), length of femorogenu IV (L11), length of tibiotarsus IV (L12), length of seta *v"* on tibiotarsus IV (L13), length of seta *tc”* on tibiotarsus IV (L14), gnathosoma length (L15), gnathosoma width (L16), and length of claw I (L17). For males, the following 13 criteria were considered: total length (L1), idiosome length without genital capsule (L2), idiosome length with genital capsule (L3), idiosome width (L4), distance between trochanters I (L5), distance between trochanters II (L6), distance between trochanters III (L7), distance between trochanters IV (L8), length of trochanter IV (L9), length of femorogenu IV (L10), length of tibiotarsus IV (L11), gnathosoma length (L12), gnathosoma width (L13) [Fig 1].

Photomicrographs (8000 in total for 88 mounted specimens) were captured under a phase-contrast microscope (Carl Zeiss Axio Imager A1 at 100x magnification) equipped with a Carl Zeiss AxioCam ERc 5s microscope digital camera. For each individual specimen, a single in-focus composite image with extended depth of field was created by stacking the corresponding series of photomicrographs captured at different focus distances with the software package Helicon Focus (version 6.7.1 Pro). Finally, parameter measurements were taken using the ImageJ software (version 1.5.3) [26].

Biometric parameter differences between the two *P*. *latus* populations were determined by assessing the assumption of normality, followed by a comparison of means with one-way and two-way ANOVA (confidence level of 95%). Moreover, multivariate canonical discriminant analysis was applied to discriminate the different populations and to determine which biometric parameters contributed more to the interpopulation differences of our study. Statistical analyses were performed with the R Project software package (version 4.0.3) [27].

A qualitative analysis by scanning electron microscopy (SEM) was performed on 20 females and 20 males for both populations of the present study and was based on the differences described by De Coss [16]. SEM observations of the mites were conducted at the *Laboratorio de Microscopio Electronico de Barrido* of *El Colegio de la Frontera Sur* in Tapachula, Chiapas.

The mite specimens were processed according to the laboratory’s standard operating protocol: after fixing the samples in 70% alcohol, they were washed with distilled water in an ultrasonic cleaning bath (Branson 1510; three times, 5 min per wash) to remove possible superficial dirt particles. Next, the samples were dehydrated in an increasing series of ethanol concentrations: 80%, 90%, and 100% each for 30 min. Then, samples were dried using hexamethyldisilazane 99.9% (twice; the first time for 30 minutes, and the second until evaporation), after which they were placed on cylindrical aluminium mounting stubs with conductive carbon tape coated with gold/palladium (70 nm thickness, applied with a Denton Vacuum Desk II sputter coater). The mite samples were then observed under a TOPCON SM-510 SEM.

### Molecular and phylogenetic study

#### DNA extraction

Five mites from the population collected in Guanajuato and six from Chiapas were randomly selected under a dissecting microscope (Carl Zeiss Stemi 2000C at 40x) and individually transferred into 1.5 mL Eppendorf tubes.

Genomic DNA was extracted using a modified protocol described by Montero-Pau et al. [28]: 50 μL of alkaline lysis solution (25 mM NaOH, 0.2 mM EDTA, pH 12). And 3.5 μL proteinase K solution (1.30 mg/mL) were added to the sample. The mixture was then incubated at 55°C for 3 h, chilled on ice for 5 min, and mechanically lysed by vortexing with glass beads. After centrifugation (10 s at 2000 g), 50 μL neutralizing solution (40 Mm Tris-HCI, pH 5) was added and the resulting mixture was vortexed and again centrifugated (10 s at 2000 g). The supernatant was then transferred to a new tube (1.5 mL), followed by precipitation with 200 μL absolute alcohol and centrifugation (4°C, 10 min at 16000 g). The supernatant was removed whilst taking care not to disturb the pellet of genomic DNA at the bottom of the tube. The pellet was washed by adding 200 μL 70% ethanol and centrifugation as before. The supernatant was then removed and the pellet dried at 70°C for 10 min before being resuspended in 20 μL injectable water.

#### Optimization of the polymerase chain reaction (PCR) for cytochrome c oxidase subunit I gene of mitochondrial DNA (*mtDNA COI*)

The DNA was amplified using the oligonucleotide primer set LCO1490 (5’GGTCAACAAATCATAAAGATATTGG3’) and HCO2198 (5’TAAACTTCAGGGTGACCAAAAAATCA3’) designed by Folmer et al. [29]. PCR conditions were as follows: an initial denaturation step at 95°C for 5 min, 30 cycles of denaturation at 95°C for 30 s, annealing at either 40°C or 48°C for 40 s, and final extension for 1 min at 72°C [30]. The PCR amplification reaction of COI was set up using PCR Master Mix (2x) (Promega, Part# 9PIM750) in a 30 μL reaction according to manufacturer protocol. Amplified products were visualized on a 1% agarose gel using SYBR Green. Finally, obtained PCR products were purified using a commercially available kit (Clean & Concentrator, ZYMO Research) according to the manufacturer’s instructions and sent to Macrogen (Seoul, South Korea) for sequencing.

#### Bioinformatic analysis

The generated amplicon was subjected to a BLAST (Basic Local Alignment Search Tool; www.ncbi.nlm.nih.gov/BLAST/) nucleotide sequence homology search against GenBank (www.ncbi.nlm.nih.gov) [31], and alignment of the sequences in question was performed using ClustalX (www.clustal.org) [32] as implemented in MEGA7. An uncorrected pairwise genetic distance matrix was computed with MEGA7 (version 7.0, Molecular Evolutionary Genetics Analysis; https://www.megasoftware.net/) [33] including our sequences and the only overlapping *P*. *latus* sequence available in GenBank (Table 1), from an individual collected in India and MEGA7 (version 7.0, Molecular Evolutionary Genetics) [33]. A neighbor-joining phylogenetic tree was constructed at the family level (Tarsonemidae) based on sequences available in GenBank (Table 1), two sequences of *P*. *latus* generated in this study (one sequence from the Chiapas population and one from Guanajuato), and an outgroup (family Podapolipidae). The statistical robustness of the phylogenetic tree nodes was assessed by bootstrap resampling analysis (1000 replicates). The COI sequences identified in this study were deposited in GenBank and assigned accession numbers MZ750953.1 to MZ750952.1 (Table 1).

**Table 1:** List of species and GenBank accession numbers (family Tarsonemidae).

Family	Identified species	Accession number
Tarsonemidae	*Polyphagotarsonemus latus* India	KM580507.1
	*Polyphagotarsonemus latus* Chiapas	MZ750953.1
	*Polyphagotarsonemus latus* Guanajuato	MZ750952.1
	Tarsonemidae gen	KY922410.1
	*Acarapis dorsalis*	GQ916567.1
	*Acarapis externus*	GQ916566.1
	*Acarapis woodi*	FJ603296.1
	*Phytonemus pallidus*	HQ694559.1
Podapolipidae	*Locustacarus buchneri*	MW077512.1

## Results

### Morphological and biometric study

**Fig 2:**
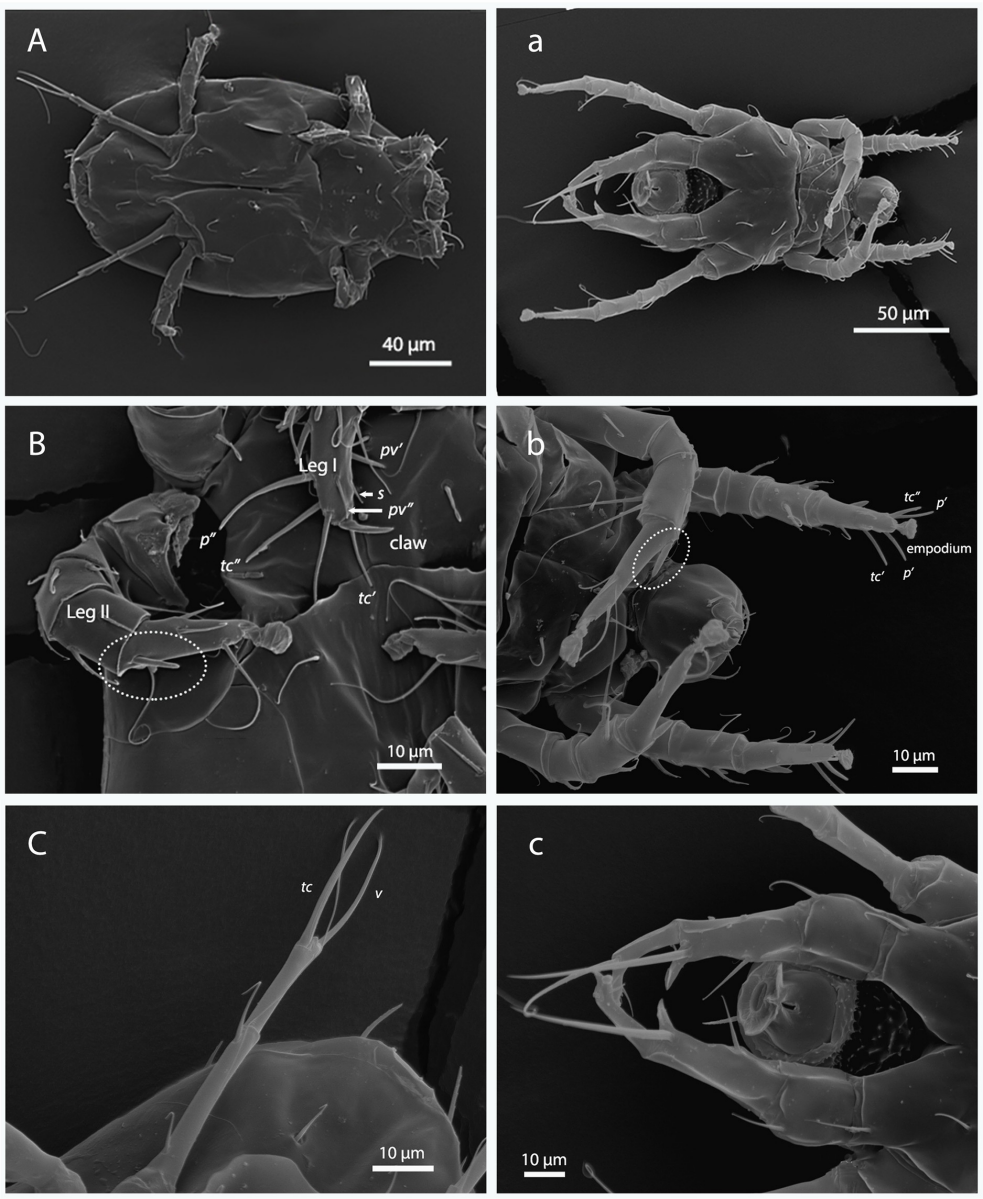
Morphology of female (A-C) and male (a-c) *Polyphagotarsonemus latus*. A-a) ventral panoramic view; B-b) chaetotaxy of tibiotarsus of Leg I [eupathidium *p´* (anteroproral), *p”* (posteroproral), *tc´* (anterotectal), and *tc”* (posterotectal); setae *pv´* (anteroprimiventral), *pv”* (posteroprimiventral) and *s* (subunguinal)] and solenidion of Leg II (within the ellipse); C-c) Leg IV [setae *v"* (posteroventral) and *tc"* (posterotectal)].

The mites collected in the two regions of this study were identified as *Polyphagotarsonemus latus* through the morphological criteria established by Lindquist [15] and Walter et al. [25]. Their taxonomic identification was verified by acarologist Gabriel Otero Colina of the *Colegio de Postgraduados* in Montecillo, Mexico. As can be seen in Fig 2A-a, the examined mites in this research showed sexual dimorphism: males were smaller than females (average of 169.79 μm and 194.12 μm, respectively) and exhibit a modification in legs III and IV (longer and smaller) which enables them to carry the female. They also show a genital capsule in which they can transport female pupae for mating in the adult stage. Length and width of the idiosome was similar for both sexes (Tables 2 and 3).

**Table 2:** Comparison of averages of biometric parameters in female specimens of *Polyphagotarsonemus latus* collected in Chiapas and Guanajuato.

key code	Biometric parameter	Chiapas	Guanajuato	P-value
L1	Total length	203.76 ± 2.52	184.49 ± 4.47	0.000392***
L2	Idiosome width	100.05 ± 2.28	89.18 ± 1.41	0.000254***
L3	Idiosome length	176.34 ± 2.38	158.54 ± 4.21	0.000492***
L4	Distance between trochanters I	31.97 ± 1.27	24.42 ± 0.83	0.0000133***
L5	Distance between trochanters II	51.50 ± 1.04	42.88 ± 1.21	0.00000222***
L6	Distance between trochanters III	65.66 ± 0.79	63.16 ± 0.76	0.0282*
L7	Length of trochanter III	28.84 ± 0.25	28.58 ± 0.43	0.594
L8	Length of femorogenu III	23.82 ± 0.21	23.81 ± 0.25	0.958
L9	Distance between trochanters IV	48.31 ± 0.70	50.99 ± 0.92	0.0241*
L10	Length of trochanter IV	12.17 ± 0.29	11.47 ± 0.28	0.0955
L11	Length of femorogenu IV	30.25 ± 0.44	31.57 ± 0.35	0.0255*
L12	Length of tibiotarsus IV	21.33 ± 0.36	19.88 ± 0.30	0.00437**
L13	Length of seta *v"* on tibiotarsus IV	23.70 ± 0.47	21.22 ± 0.49	0.00076***
L14	Length of seta *tc”* on tibiotarsus IV	61.11 ± 3.05	63.58 ± 3.31	0.586
L15	Gnathosoma length	30.53 ± 0.55	27.14 ± 0.64	0.000247***
L16	Gnathosoma width	28.81 ± 0.36	28.39 ± 0.36	0.423
L17	Length of claw I	9.51 ± 0.22	7.63 ± 0.17	0.0000000392***

Highly significant differences

***; significant differences

**; slightly significant differences

* (α = 0.05).

All measurements in μm; standard deviation included.

**Table 3:** Comparison of averages of biometric parameters in male specimens of *Polyphagotarsonemus latus* collected in Chiapas and Guanajuato.

key code	Biometric parameter	Chiapas	Guanajuato	P-value
L1	Total length	164.33 ± 2.7	175.25 ± 2.36	0.00515**
L2	Idiosome length without genital capsule	93.26 ± 2.04	101.92 ± 2.84	0.0158*
L3	Idiosome length with genital capsule	138.23 ± 2.51	148.65 ± 2.64	0.00714**
L4	Idiosome width	67.38 ± 1.87	75.92 ± 2.53	0.00866**
L5	Distance between trochanters I	33.75 ± 1.51	30.83 ± 1.34	0.167
L6	Distance between trochanters II	48.10 ± 2.36	54.20 ± 2.46	0.0838
L7	Distance between trochanters III	59.28 ± 1.02	57.57 ± 1.15	0.274
L8	Distance between trochanters IV	56.29 ± 1.35	58.63 ± 1.61	0.269
L9	Length of trochanter IV	19.10 ± 0.37	20.72 ± 0.57	0.0199*
L10	Length of femorogenu IV	29.35 ± 0.31	32.96 ± 0.36	0.00000000458***
L11	Length of tibiotarsus IV	25.17 ± 0.57	27.69 ± 0.5	0.00714**
L12	Gnathosoma length	27.79 ± 0.57	27.74 ± 0.77	0.965
L13	Gnathosoma width	28.01 ± 0.45	27.80 ± 0.38	0.736

Highly significant differences

***; significant differences

**; slightly significant differences

* (α = 0.05).

All measurements in μm; standard deviation included.

The mite gnathosoma has a capsular shape wider than long, as could be observed in male specimens from both populations and females from Guanajuato, but not in females from Chiapas (30.53 ± 0.55 μm long and 28.81 ± 7.63 μm wide) (Tables 2 and 3).

In females, Leg I was observed to have a large sesille and slightly curved claw (Fig 2C) whose average length was higher in specimens from Chiapas (9.51 ± 0.22 μm) than in those from Guanajuato (7.63 ± 0.17 μm) (Table 2). Setae *p’* (anteroproral), *p"* (posteroproral), *tc’* (anterotectal), *tc"* (posterotectal), *pv’* (anteroprimiventral), *pv"* (posteroprimiventral), and *s* (subunguinal) can be distinguished in Leg I (tibiotarsus segment). Seta *s* setiform is smaller than seta *pv"*, while seta *pv" i*s larger and thicker than *s*. Likewise, seta *pv’* is thicker than *pv"*, and seta *tc’* is shorter than *tc”* (Fig 2B). Legs II and III exhibit an empodium that arises from the distal part, and in Leg III a fusion of femur and genu can be observed (femorogenu). The average femorgenulength was similar for specimens from both populations (23.82 ± 0.21 μm and 23.81 ± 0.25 μm for Guanajuato and Chiapas, respectively). Leg IV has a thin, elongated shape and can be subdivided in trochanter, femorogenu and tibiotarsus (Fig 2C). The average lengths of these segments are similar for both populations. Two thin setae can be detected in the tibiotarsus; seta *v"* is shorter than *tc"* (Table 2).

Males, on the other hand, lack the sessile claw, but instead have an empodium on the tarsus of Leg I, and also have a chemosensory seta. Seta *p*" is smaller than *p*’ and *tc"* is larger than *tc’* (Fig 2b). A solenidion can be distinguished on Leg II, which is similar to but thicker than the female solenidion. Legs IV are thicker and with a curved shape. They can be subdivided in three segments, viz. trochanter, femorogenu and tibiotarsus (Fig 2c). The average length of the trochanter is similar for both populations (Fig 2c and Table 3). In the case of the femorogenu, the average lengths are higher in specimens from Guanajuato (29.35 ± 0.31 μm) than in those from Chiapas (32.96 ± 0.36 μm) (Table 2 and Fig 2c).

Biometric analysis of the parameters for female *P*. *latus* mites indicated highly significant differences in the following variables: total length (L1), idiosome width (L2), idiosome length (L3), distance between trochanters I (L4), distance between trochanters II (L5), distance between trochanters III (L6), distance between trochanters IV (L9), length of femorogenu IV (L11), length of tibiotarsus IV (L12), length of seta *v"* on tibiotarsus IV (L13), gnathosoma length (L15), and length of claw I (L17) for mites from both populations. Specimens from Chiapas were larger. The remaining variables (L7, L8, L10, L14 and L16) were similar for both regions (Table 2). An analysis of variance (ANOVA) indicated a significant difference between the investigated populations, with a significant interaction between both factors (F = 7.45; df = 16, 782; P < 0.0001).

**Fig 3:**
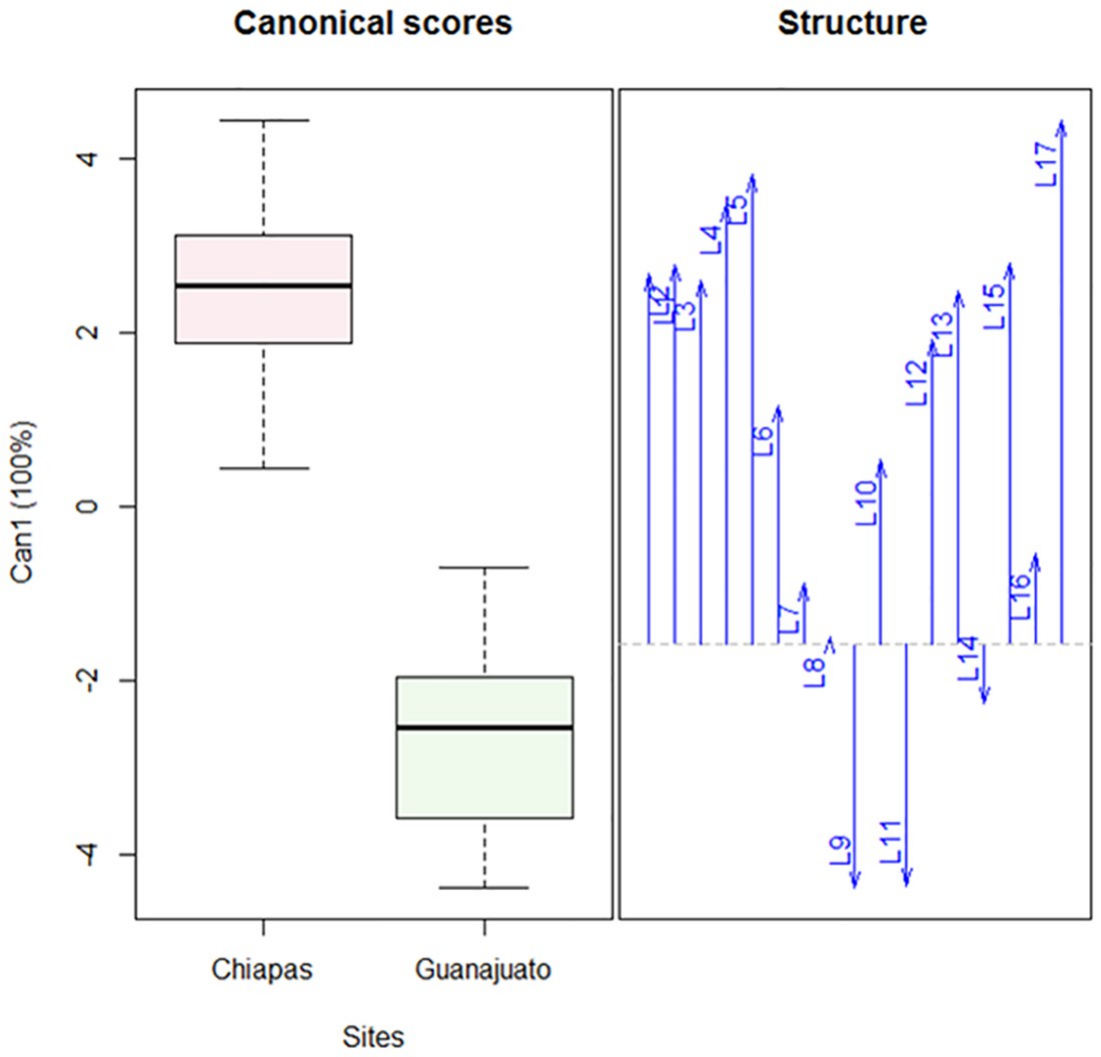
Canonical discriminant analysis of the morphological variables of *Polyphagotarsonemus latus* (females). The box-and-whisker plot shows a difference between both populations; arrows indicate the correlation: L9, L11 and L14 are negatively correlated and affect the separation between the populations. The key to the abbreviations can be found in Materials and methods (Morphological and biometric study).

Applying canonical discriminant analysis to the same data revealed a significant separation for females between the studied populations (Pillai test: F = 12.77; 1,17; P<0.0001) (Fig 3). In general, the measured lengths of the different parameters are positively correlated, with the exception of the distance between trochanters IV (L9), length of femorogenu IV (L11), and length of seta *tc”* on tibiotarsus IV (L14). These are negatively correlated to other lengths and predominate in the population from Guanajuato (Fig 3).

The biometric analysis of the parameters for male broad mites showed variability. Total length (L1), idiosome length without and with genital capsule(L2 and L3), idiosome width (L4), length of trochanter IV (L9), length of femorogenu IV (L10) and length of tibiotarsus IV (L11) were observed to be higher in the population from Guanajuato than in the population from Chiapas. The remaining parameters (L5- L8, L12 and L13) were similar for both regions (Table 3). An analysis of variance revealed significant differences between both populations, with a significant interaction between both factors (F = 2.84; df = 12, 494; P < 0.0001).

**Fig 4:**
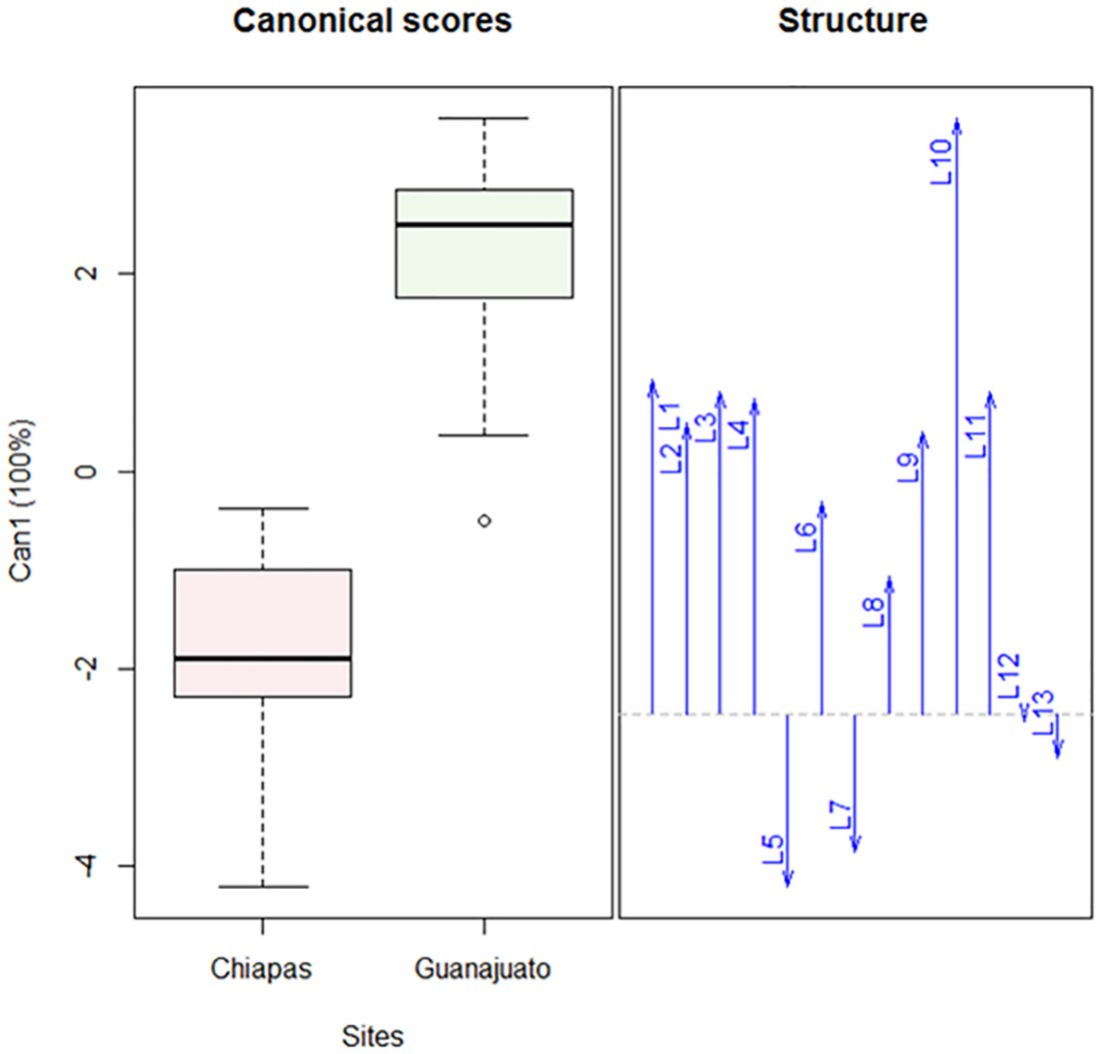
Canonical discriminant analysis of the morphological variables of *Polyphagotarsonemus latus* (males). The box-and-whisker plot shows a difference between both populations; arrows indicate the correlation: L5, L7 and L13 are negatively correlated and affect the separation between the populations. The key to the abbreviations can be found in Materials and methods (Morphological and biometric study).

Canonical discriminant analysis indicated a significant separation for males between both population (Pillai test: F = 8.53; 1,13; P<0.0001) (Fig 4). The distance between trochanters I (L5), distance between trochanters III (L7) and gnathosoma width (L13) are negatively correlated with the other parameters (L1-L4, L6, L8-L12).

### Molecular and phylogenetic study

This study analysed the nucleotide sequences of the COI gene obtained from 11 individuals (five collected from a population in Guanajuato and six in Chiapas) of *P*. *latus*. A homology search against the GenBank database revealed that the sequences from the Guanajuato and Chiapas populations showed 83.0–86.41% and 99.26–99.52% similarity, respectively, with accession number KM580507.1 (*Polyphagotarsonemus latus* from India).

The average fragment size was 447 bp (range 431–469 bp). A review of the nucleotide base composition revealed an elevated frequency of thymine (T) and adenine (A) (43.2% and 27.9%, respectively) compared to cytosine (C) and guanine (G) (14.7% and 14.2%, respectively).

The aligned sequences of the two Mexican populations from this study and the sequence obtained from GenBank KM580507.1 comprised 408 characters—excluding gaps (insertions/deletions)—of which 347 were constant and 61 variable sites. The latter consisted of 61 singletons and 0 parsimony-informative sites. It is important to mention, however, that no nucleotide differences (intraspecific) were observed between the two populations of the present investigation. Translation of all sequences into amino acids did not reveal any stop codons.

Genetic distances (p-distance) calculated between our samples and the sequence retrieved from Genbank shows that the mite population from Chiapas has the highest genetic similarity with the population from India (KM580507.1; p-distance = 0.010), whereas the population from Guanajuato is genetically more distant (0.191) (Table 4). From these data, it can be deduced that the mites from Chiapas, Guanajuato and India likely represent differentiated populations.

**Table 4:** Pairwise genetic distances. Calculations based on sequenced COI fragment.

	(MZ750953.1) Chiapas	(MZ750952.1) Guanajuato
**(MZ750952.1) Guanajuato**	0.191	
**(KM580507.1) India**	0.010	0.208

**Fig 5:**
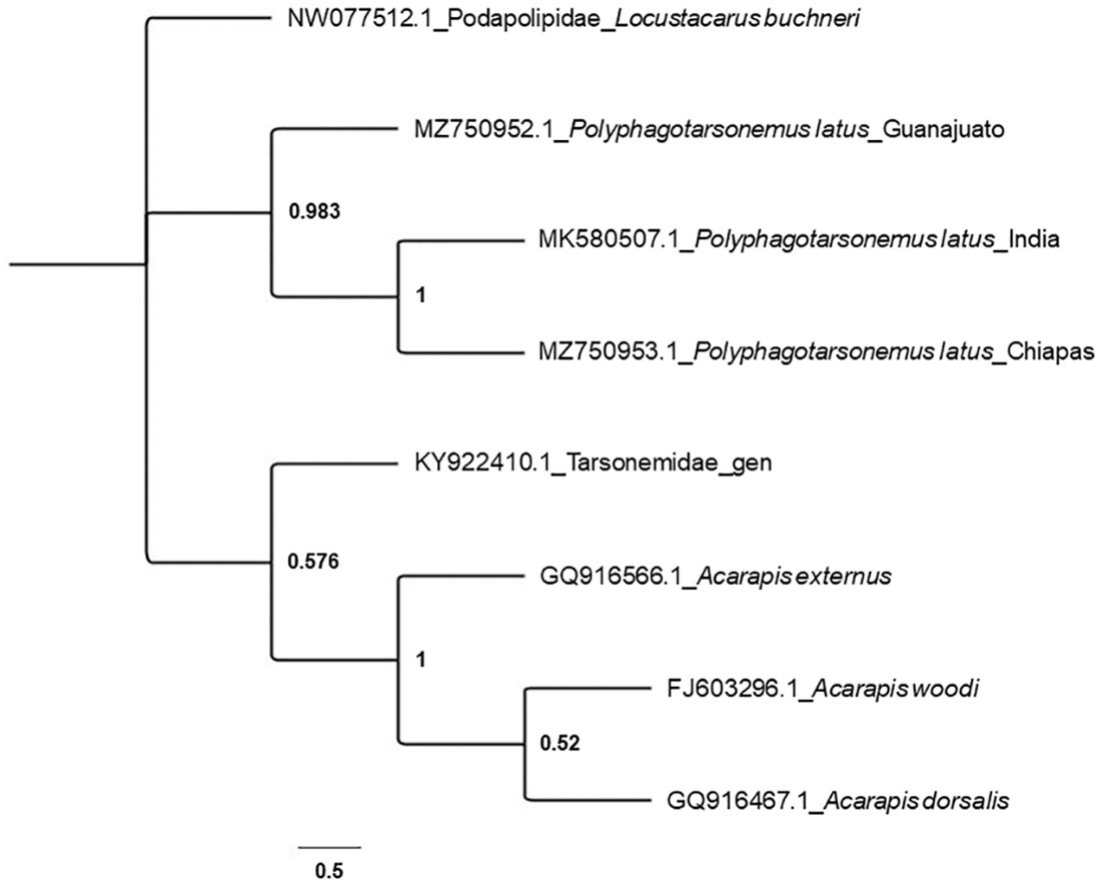
Consensus tree of the family Tarsonemidae represented in the Genbank. The tree was constructed based on sequences of mitochondrial COI fragments, with maximum likelihood and based on general time reversible model. The bootstrap consensus tree was inferred from 1000 replicates and used the family Podapolipidae as outgroup.

A phylogenetic tree was constructed by using Tarsonemidae sequences available in the GenBank database and selecting a sequence from the family Podapolipidae as an outgroup. The tree illustrates that the individuals of the current study are related to the rest of the family Tarsonemidae, and also reveals two distinct clades. These results are supported by Lindquist’s (1986) morphological findings. *Acarapis* spp and *Polyphagotarsonemus* latus belong to two different subfamilies. The first clade contains the genus *Acarapis* and another sequence labelled only as ‘Tarsonemidae’ and another clade including *Polyphagotarsonemus latus* where the sequence from Chiapas (this study) is more closely related to the sequence from India than to the sequence from Guanajuato (this study). These relationships are supported by the previously calculated genetic distances values (Fig 5).

## Discussion

The qualitative comparison of male and female *P*. *latus* morphology revealed a clear sexual dimorphism, as has previously been described by Karl [8]. The empodial claw on tarsus I of the female and the buttonlike claw of the male can be considered typical characteristics of the genus *Polyphagotarsonemus* [34,35]. According to Schaarschmidt [36], this genus has a gnathosoma with an extended shape, which in our study was observed in females from Chiapas, but not in females from Guanajuato nor in males of either population (Tables 2 and 3)—in contrast, they were typified by a gnathosoma that is wider than it is long.

Comparison of the tarsus I chaetotaxy of broad mite females according to the criteria set out for specimens from the Soconusco region (Chiapas) by De Coss [16], revealed that unguinal setae *u’-u"* are not present in Mexican specimens. Also, subunguinal seta *s* was observed to be setiform, smaller than *pv"* and *pv’*, and relatively thick compared to *P*. *latus* specimens from Brazil, which were described as having a slightly thick seta *pv’*, setiform s of nearly the same size as *pv"*, and with imperceptible u’-u" [15]. Tarsus I, moreover, has a sessile claw about halfway along its length. This claw is larger in specimens from Chiapas than those from Guanajuato (9.51 ± 0.22 μm and 7.63 ± 0.17 μm, respectively) (Table 2). The tarsal claws of broad mites from Guanajuato were similar in length to the tarsal claws of *P*. *latus* from Costa Rica (8.85 μm, as reported by Ochoa et al. [18]). Their lengths, moreover, were also comparable to those reported by De Coss (7.67 μm) [16].

A variation of 70.58% between the females of both Mexican populations was observed in the biometric parameters (Chiapas specimens are larger). The total length of the specimens from Guanajuato corresponded to the length of German broad mites reported by Karl (184.9 μm) [8]. The German specimens, however, were reared in the laboratory and Ramos et al. [17] remark that laboratory-reared broad mites are typically smaller than specimens collected from the wild. The wild-caught broad mites from Cuba (246 μm) described by Ramos et al. were also larger than the Mexican specimens of the present study (Chiapas **♀**: 203.76 ± 2.52 μm; Guanajuato **♀**: 184.49 ± 4.47 μm), even though the latter were collected from the wild as well. The distance between trochanters I-III, as well as width and length of the idiosome were smaller than those reported for Cuban specimens [17].

A significant variation between the females of both populations could be deduced from the analysis of variation (F = 86.21; df = 1, 782; P < 0.0001) as well as the canonical discriminant analysis (Pillai test: F = 12.77; 1, 17; P < 0.0001).

A variation of 53.84% between the broad mite males was observed in the 13 biometric parameters (Table 3). The average of the total length (Chiapas ♂: 164.33 ± 2.7 μm; Guanajuato ♂: 175.25 ± 2.36 μm) is comparable to specimens from Germany (141.4 μm) [8] and Costa Rica (184.05 μm) [18], whereas specimens from Cuba are larger (276 μm) [17]. No variation has been observed in the idiosome length with papillae for specimens from the aforementioned countries, although variation regarding specimens described by De Coss (from Chiapas, Mexico [16]) has been detected in the distance between trochanters II. Similarly, variation regarding the Cuban population was observed in the length of femorogenu IV [17].

Analysis of variation (F = 25.46; df = 1, 494; P < 0.0001) as well as canonical discriminant analysis (Pillai test: F = 8.53; 1, 13; P < 0.0001) revealed a significant variation between the males of both Mexican populations.

The genetic and molecular characteristics of *P*. *latus* have not been extensively studied yet. Analysis of the mtDNA COI fragment reveals considerable variation between *Polyphagotarsonemus* sp. mites. They display an AT skew (calculated from the data in Table 4)—a typical characteristic of the COI gene of arthropods—similar to that of other insect and mite taxa [37,38]. The genetic distance between the population from Chiapas and India KM580507.1 was smaller compared to the population from Guanajuato and KM580507.1 (Table 4). The phylogenetic tree supports the separation between the Mexican populations of the present study and *Acarapis*, a genus of parasitic mites of honeybees that may adversely affect honey production [39] and the only other member of the family Tarsonomidae represented in Genbank. The tree also shows a clear separation of *P*. *latus* collected in Chiapas and India from those in Guanajuato. The populations are clearly differentiated and may represent a species-complex, as previously noted by Lindquist [15] in his redefinition and redescription of the taxonomical position of *P*. *latus*. The populations from Chiapas and Guanajuato present important qualitative, quantitative and molecular differences between themselves as well as previously investigated populations. It is important to recognize that environmental variables (temperature, humidity), hosts, and other parameters, can promote morphological changes—phenotypic plasticity, polymorphism, race formation, and even speciation [40]. The population from Guanajuato was collected from a cultivated host (commercial cultivation site), where the mites might have been exposed to selection pressure (development of resistance) from the use of agrochemicals and which might have induced changes in their genotypes.

It is important to study in more detail this group of mites of worldwide economic importance. There are marked morphological and molecular variations in populations that are geographically separated and that come from different hosts (wild and cultivated, as is the case of this study). Therefore, it is necessary to expand the analysis to include specimens from other countries (including Sri Lanka, its center of origin) and different ranges of hosts, and also to explain the phylogenetic differentiation observed in this study.

## Conclusion

The precise identification of mite species is necessary for our comprehension and interpretation of evolutionary processes, ecological diversity, and to develop methods of phytophagous mite control. However, morphological identification of mites can be challenging because many traits exhibit phenotypic plasticity and lack identification keys. On the other hand, the sequencing of the COI gene could provide an important and powerful tool for species identification, but the use of a single (mitochondrial) gene may not be adequate. An integrated approach is called for to focus on combining nuclear and mitochondrial genes, morphological characteristics and ecological information.

Our data show that *P*. *latus* populations from Chiapas and from Guanajuato exhibit significant differences in their morphology and variation in their nucleotide sequences. The latter also differ from sequences from Indian specimens available in GenBank.

In our investigation, we implemented a rapid and efficient molecular diagnostic tool for the monitoring of phytophagous mites. New molecular taxonomy data can be visualized as an aid to the control of *Polyphagotarsonemus* sp. Our study incorporates useful information technologies, such as bioinformatics, in the diagnosis of phytophagous mites.

## Supporting information

S1 Dataset Analysis of the morphological and biometric data of two Mexican populations of mites. (XLSX)
